# Systemic Deficiency of PTEN Accelerates Breast Cancer Growth and Metastasis

**DOI:** 10.3389/fonc.2022.825484

**Published:** 2022-03-18

**Authors:** Jing Chen, Jingjing Sun, Qunfeng Wang, Yanze Du, Jie Cheng, Juan Yi, Bei Xie, Suya Jin, Gang Chen, Lina Wang, Xiaoyuan Wang, Hulai Wei

**Affiliations:** ^1^ Key Laboratory of Preclinical Study for New Drugs of Gansu Province, School of Basic Medical Sciences, Lanzhou University, Lanzhou, China; ^2^ Department of Clinical Laboratory, The First Hospital of Lanzhou University, Lanzhou, Gansu, China; ^3^ Department of Thoracic Surgery, Gansu Provincial Cancer Hospital, Lanzhou, China

**Keywords:** PTEN, 4T1 cell, multiplication, invasion, metastasis

## Abstract

Mutation or loss of the tumor suppressor gene *PTEN* or its functional status in tumor stromal cells may affect tumor occurrence, development, invasion, and metastasis, in which, however, the role of overall low PTEN expression, mutation, or deletion in the tumor-bearing host has rarely been reported. Breast cancer is a common highly invasive metastatic tumor. We therefore treated mouse breast cancer 4T1 cells with the specific PTEN inhibitor VO-OHpic to study the effects of PTEN suppression or deletion on malignant behavior *in vivo* and *in vitro*. VO-OHpic effectively inhibited PTEN gene/protein expression in 4T1 cells, accelerated cell proliferation, and enhanced cell migration and invasion. We also transplanted 4T1 cells with VO-OHpic-inhibited PTEN into mice to create orthotopic and metastatic breast cancer models. The proliferation of 4T1 cells in mouse mammary gland was increased and distant metastasis was enhanced, with metastatic foci in the lung, liver, and intestinal tract. In addition, injection of mice with VO-OHpic to inhibit PTEN in the overall microenvironment accelerated the proliferation of transplanted 4T1 cells and enhanced distant metastasis and the formation of metastatic tumors. Metastatic foci formed in the lung, liver, intestine, thymus, and brain, and PTEN levels in the organ/tissues were negatively associated with the formation of metastatic foci. Similarly, inoculation of PTEN-deficient 4T1 cells into systemic PTEN-inhibited mice further enhanced the orthotopic growth and distant metastasis of 4T1 breast cancer. VO-OHpic inhibition of PTEN in 4T1 cells was also associated with significantly increased phosphorylation of Akt and phosphoinositide 3-kinase (PI3K), suggesting that inhibition of PTEN could activate the PI3K-Akt pathway, as a key signaling pathway regulating cell proliferation and death. These results confirmed that functional loss or deletion of the tumor suppressor gene PTEN significantly enhanced the proliferation, invasion, and metastasis of 4T1 cells. Systemic decrease or deletion of PTEN in the organism or organ/tissue microenvironment was conducive to the proliferation of breast cancer cells *in situ* and distant metastasis. These results suggest that, as well the PTEN in cancer cells the systemic microenvironment PTEN intensely mediates the proliferation, invasion and metastasis of mouse breast cancer cells *via* regulating the PI3K-Akt signaling pathway.

## Introduction

Breast cancer is the most common malignant tumor in women, with the highest morbidity and mortality rates (1, 2). Its incidence and mortality are currently increasing year by year, with a trend towards younger patients (1–3). According to the latest data released by the China National Cancer Center in 2019, breast cancer accounted for 7.74% of all malignant tumors and 2.99% of the total mortality in China in 2015. The incidence of breast cancer in China (7.7%) is higher than that worldwide (5.8%), with about 304,000 new cases each year (4). Invasion and distant metastasis are the main causes of death due to breast cancer, accounting for >90% of breast cancer-related deaths. The main target organs of breast cancer metastasis are the lungs, liver, bone, lymph nodes, and brain (4–8). Numerous studies have shown that breast cancer metastatic ability depends not only on the characteristics of the cancer itself, but also on the tumor microenvironment and interactions between tumor cells and the tumor microenvironment, with the target organ microenvironment having a strong influence on the fate of the cancer cells and the formation of metastatic carcinoma (7–11). We therefore propose that the primary tumor microenvironment regulates the proliferation, invasion, and metastatic characteristics of breast cancer cells, while the fate of those cells depends on the microenvironment of the distant metastatic target organs. Invasion and metastasis of breast cancer cells currently presents a problem in treating breast cancer patients and inhibiting or blocking these processes is key to the effective prevention and treatment of breast cancer.

The invasion and metastasis of breast cancer cells is a multi-gene, multi-stage process involving the activation of oncogene mutations and inactivation of tumor suppressor genes (7–9). Phosphatase and tensin homolog (*PTEN*) is a tumor suppressor gene with dual protein phosphatase and lipid phosphatase activities discovered in 1997, which is second only to *P53* (10, 11). *PTEN* is widely involved in cell proliferation, differentiation, adhesion, migration, metastasis, and apoptosis, as well as in the cell cycle, energy metabolism, genome stability, and other processes (10–14). Previous studies have confirmed that *PTEN* mutation and functional loss play a key role in the occurrence, development, and metastasis of malignant tumors (13–15).

*PTEN* abnormalities in breast cancer cells are mainly caused by deletion mutation, abnormal promoter DNA methylation, and abnormal degradation or functional loss of PTEN protein expression (10, 13–18). Loss of heterozygosity of *PTEN* has been reported in about 40%–50% of breast cancer patients, and about 5%–10% of patients with breast cancer have *PTEN* mutations, most commonly frameshift mutations, resulting in PTEN functional loss (13, 15, 18). *PTEN* inactivation is mainly attributed to somatic mutations, including missense and nonsense mutations, single- or double-allele deletions at *PTEN* gene loci, resulting in PTEN protein degradation and post-translational changes as a result of epigenetic silencing *via* promoter methylation (13–17, 19). Similarly, PTEN function may be regulated by post-translational modifications, such as phosphorylation, acetylation, oxidation, monoubiquitination, and polyubiquitination (10–13, 15–20). Most studies on the role of PTEN in the invasion and metastasis of breast cancer and other malignant tumors have focused on the functional changes of PTEN in the tumor cells themselves and have ignored the effects on the overall function of tumor-bearing hosts, and the effects of target organ-specific PTEN on tumor cell invasion, distant metastasis, and colonization (21–23).

The embryo-lethality of homozygous *PTEN* knockout makes it difficult to prepare a *PTEN* full-gene knockout mouse model, and only conditional or heterozygous knockouts models can be produced (23, 24), making it difficult to analyze the effect of the overall PTEN status of tumor-bearing hosts on breast cancer proliferation and metastasis. Current studies on the relationship between PTEN in the tumor microenvironment and the invasion and metastasis of breast cancer and other malignant tumors are mostly limited to examining the effect of PTEN expression in the primary tumor microenvironment on the proliferation, invasion, and metastasis of tumor cells *in situ* (9–11, 18, 22–25). However, few studies have considered the effect of the host itself, especially regarding the functional state of PTEN in the microenvironment of the metastatic target organ, on the ability of the metastatic breast cancer cells to colonize, proliferate, and form metastatic foci in the organ, and the mutual adaptation of cells in the target organ microenvironment and metastatic breast cancer cells.

We therefore investigated the effects of the PTEN-specific inhibitor VO-OHpic in BALB/c mice and in the derived breast cancer 4T1 cells, to determine the effects of PTEN on the proliferation, migration, and invasion of 4T1 cells and their ability to form distant metastases *in vivo*. We also investigated the effects of PTEN levels in the host mouse or target organ microenvironment on the colonization and survival of metastatic breast cancer cells in the target organs and on the formation of metastatic cancers. We aimed to clarify the mechanism by which the functional status of PTEN in the whole host/metastatic organ microenvironment determines the fate of metastatic breast cancer cells, and to explore strategies to reduce the invasion and metastasis of breast cancer by enhancing PTEN levels in tumor-bearing organisms.

## Materials and Methods

### Reagents and Cells

Dulbecco’s Modified Eagle Medium (Gibco BRL, MD, USA), fetal bovine serum (BI Biotechnology, Kibbutz Beit Haemek, Israel), TRIzol reagent (Invitrogen Penrose, Auckland, New Zealand), RIPA protein lysate BCA protein concentration assay kits, and Crystal violet solution were all from Solebro (Beijing, China). Rabbit anti-PTEN polyclonal antibody, mouse anti-phospho (Ser-473 cat no: 3257-100)-, rabbit anti-Akt polyclonal antibody (3247–100) were purchased from Biovision Inc. (CA, USA). Rabbit anti- PI3 Kinase p85 (Tyr-467, cat no: GTX132597)-, PI3 Kinase p85 alpha (GTX111068) polyclonal antibody were from GeneTex (Texas, USA). Color pre-dyed Marker was obtained from Thermo Scientific (MA, USA), Prime Script™ RT Reagent Kit with gDNA Eraser and SYBR Premix Ex Taq were from TaKaRa Bio (Dalian, China), and VO-OHpic and luciferin were from Sigma−Aldrich (Darmstadt, Germany).

Mouse breast cancer 4T1 cells and luciferase gene-labeled mouse breast cancer 4T1 cells (4T1-luc) were provided by the Laboratory of Medical Laboratory Zoology, School of Basic Medicine, Lanzhou University. The cells were routinely cultured in complete culture medium containing 10% fetal bovine serum.

### Xenograft Mouse Model

Balb/c mice (female, 5–6 weeks old, body weight 20 ± 2 g) maintained in a specific-pathogen-free (SPF) animal room were purchased from the Experimental Animal Center of Lanzhou University (production license number: SCXK (GAN) 2018-0002; use license number: SYXK (GAN) 2018-0002). The management, care, and ethical welfare of the experimental animals were all in accordance with the Guiding Suggestions on the Ethical Review of Experimental Animal Welfare (GB/T 35892-2018) issued by the Ministry of Science and Technology of China.

### Cell Viability Assay

#### MTT Colorimetric Assay

4T1 cells were inoculated into 96-well plates at a density of 0.6×10^5^ cells/mL, and then cultured at 37°C until they adhered to the plate. 200 or 500 nmol/L VO-OHpic were then added for 4 h. The culture medium was then replaced without VO-OHpic for a further 24–72 h, and 10 µl MTT (5 mg/mL) was added to each well for the last 4 h. After continuous culture for 4 h, 10% sodium dodecyl sulfate (SDS) 100 µl per well was added and incubated overnight at 37°C. The absorbance value (λ=570 nm) was determined by enzyme-linked immunoassay (Powerwave X plate reader Omega Bio−Tek, Inc., Norcross, GA, USA) to calculate the proliferation rate or proliferation inhibition rate of 4T1 cells.

#### Clonogenicity Assay

Normally cultured 4T1 cells were inoculated into 6-well plates at a density of 5×10^3^ cells/ml. After the cells attached to the well, 200 or 500 nmol/L VO-OHpic was added to the cells for 30 min–4 h. The supernatant was then discarded, and 2 ml complete medium was added followed by further culture for 72 h. The supernatant was discarded again, and the cells were fixed with 100% methanol for 30 min, stained with 0.1% crystal violet for 30 min, washed with water, and dried at room temperature, and cell proliferation was observed by light microscopy (original magnification, ×10).

### Real Time Quantitative Reverse Transcription-Polymerase Chain Reaction (RT-PCR)

Target cells were collected, and total RNA was extracted using TRIzol. Samples were reverse transcribed using a Prime Script™ RT Reagent Kit with gDNA Eraser (Perfect Real Time; Takara), according to the manufacturer’s instructions, with 5× gDNA Eraser Buffer to obtain the first cDNA strand. The cDNA was then amplified using a Prime Script reverse transcriptase kit (Takara Bio, Inc.) according to the following protocol: 70°C for 30 min, 37°C for 15 min, and 95°C for 5 min. For PCR, cDNA was mixed with SYBR Premix Ex Taq and subjected to 40 cycles of denaturing at 95°C for 5 s and annealing at 60°C for 30 s. The following primers for PCR were designed and synthesized by Takara Bio, Inc. and analyzed by Rotor-Gene 6.0 using the comparative domain value method: β−actin forward, 5’-TGCTCCTCCTGAGCGCAAGTA-3’ and reverse, 5′-CCACATCTGCTGGAAGGTGGA-3′; and PTEN forward, 5′-CTCCTCTACTCCATTCTTCCC-3′ and reverse, 5′-ACTCCCACCAATGAACAAAC-3′

### Cell Invasion and Migration

The ability of target cells to penetrate synthetic basement membranes was assessed using a Matrigel−Boyden chamber (BD Biosciences, NJ, USA). The transwell compartment was placed in a 24-well plate filled with complete medium and cells were collected and seeded into the wells at a density of 5×10^3^ cells per well, in serum−free medium. After incubation for 36 h, the compartment was removed, cells above the synthetic membranes were wiped off with cotton swabs, and the lower side of the compartment was fixed in 4% paraformaldehyde for 30 min–1 h, and then stained with 0.1% crystal violet at room temperature for 30 min. Cells that crossed the membranes were observed and imaged using an optical microscope (Olympus IX81, Japan). Cell migration ability was assessed as for the invasion assay, except for the absence of Matrigel.

### Cell Scratch Assay

4T1 cells were plated in 6-well plates at 0.6×10^5^ cells per well. The next day, the cells were treated with 200 or 500 nmol/L VO-OHpic, respectively, for 30 min–4 h. The medium was then replaced with complete culture medium without VO-OHpic, and a straight line was drawn across the middle of the adherent cell layer using a 200 μl pipette tip. Imaging was performed under an optical microscope at 0 h and after incubation for 24 h, to calculate the cell-migration rate.

### Cell Cycle Analysis

After treatment of 4T1 cells with 200 or 500 nmol/L VO-OHpic, respectively, for 30 min–4 h, the cells were collected, washed with phosphate-buffered saline (PBS), blended gently, and suspended in 70%–75% cold ethanol overnight at −20°C, washed with PBS, and 500 µl propidium iodide dye was added to each tube for dark staining for 30 min. The distribution of cell cycle phases was determined by flow cytometry (ACEA NovoCyte, Jiangsu, China).

### Western Blotting

Protein expression was determined by western blotting. Proteins from cells were isolated and dissolved with RIPA lysis buffer and their concentration levels were determined using the BCA method (Solarbio, Beijing, Chian). The proteins (30 μg) were subsequently separated by 10% SDS−polyacrylamide gel electrophoresis and transferred onto polyvinylidene difluoride membranes (Millipore, MA, USA), and blocked with 5% nonfat milk at room temperature for 1 h. After washing three times with PBS−Tween−20 for 5 min, the membranes were probed with primary antibodies at 4°C overnight. As for immunodetection, the membranes were incubated at room temperature with horseradish peroxidase-conjugated secondary antibody for 1 h and subsequently observed and analyzed using an Amersham Enhanced Chemiluminescence system (GE Healthcare Life Sciences, Beijing, Chian).

### Mouse Homograft Tumor Model

We established mouse orthotopic and metastatic tumor models by inoculation of 4T1-luc breast cancer cells into the breast fat pad or caudal vein of female Balb/c mice (8 weeks old, body weight 20 ± 2 g), and observed the tumor cell growth and metastasis by small-animal imaging (IVIS Lumina II, Caliper Life Science, Cold Spring Harbor, USA). To create an orthotopic model, 4T1−luc cells (2×10^6^/ml) were treated with 0, 200, or 500 nmol/L VO-OHpic, respectively, for 2 h *in vitro* to inhibit PTEN expression, and then inoculated into the breast fat pad, or mice were injected intraperitoneally with 10 or 20 μg/kg VO-OHpic, and 4T1−luc cells (2×10^6^/ml) were then inoculated directly into the fat pad. 4T1−luc cells treated as above were also injected into the tail vein of female Balb/c mice to establish a metastatic model. All the model mice were raised under SPF conditions and observed regularly.

The formation of *in situ* and metastatic tumors in the above animal models were observed using a small-animal imaging system at 7, 15, and 21 days after inoculation, respectively. The mice were sacrificed humanely at the end of the experiment, and the *in situ* tumor masses, as well as tumors in the lungs, liver, intestines, and kidneys, were isolated, and the tumors and organs were observed by imaging. Some fresh tissues were also used for RNA and protein extraction to detect the expression of related genes.

This animal experiment program was reviewed and approved by the Laboratory Animal Science and Technology Management Committee of the School of Basic Medicine of Lanzhou University (Approval Number: LZUSBMS-EAAC-2018-009), in accordance with the Ministry of Science and Technology of the People’s Republic of China on the Guiding Opinion on the Treatment of Laboratory Animals and the national standard “Laboratory Animal Welfare Ethics Review Guide” (GB/T 35892-2018).

### Immunohistochemical (IHC) Assay

The following IHCs, according to manufacturers’ protocols were performed: Freshly isolated tissues were fixed with formalin and embedded with paraffin, cut into 4–5 µm slices, and fixed on slides with a polylysine coating. The slides were deparaffinized in xylene followed by graded ethanols (100%, 95%, 70%, 50%), and finally washed in cold running water. Endogenous peroxidase was removed using 0.5% hydrogen peroxide in methanol solution, and antigen blocking was carried out using 5% bovine serum albumin, following antigen retrieval in a pressure cooker using a thermally-induced method that the slides were put into an autoclaver containing medlar buffer, heated for 5 minutes, and then cooled in cold water to repair epitopes in citrate buffer (pH 6). Slides were incubated with primary antibodies at different dilution ratios to determine the optimal concentrations. The slides were then washed and treated with a secondary antibody. Incubations with antibody (Rabbit anti-PTEN, Cell Signalling, Massachusetts, USA) was carried out in a humidified box to avoid drying. After incubation with the secondary antibodies, the slides were washed with PBS, covered with freshly prepared DAB color solution, and then washed again with water, followed by hematoxylin staining, clearing, drying, and mounting. The immunohistochemical stains were evaluated by two pathologists with consensus.

### Statistical Analysis

All data were analyzed using SPSS version 22.0 (SPSS, Inc., Chicago, IL, USA) and the statistical results were presented as mean ± standard deviation. Multi-factor comparisons between samples were performed using two−way analysis of variance followed by a Newman−Keuls *post hoc* test. All experiments were repeated at least three times. *P*<0.05 was considered to indicate a significant difference, and error bars were used to represent the standard error of the mean.

## Results

### Inhibition of PTEN Expression by VO-OHpic Increased Proliferation of 4T1 Breast Cancer Cells

**Figure 1:**
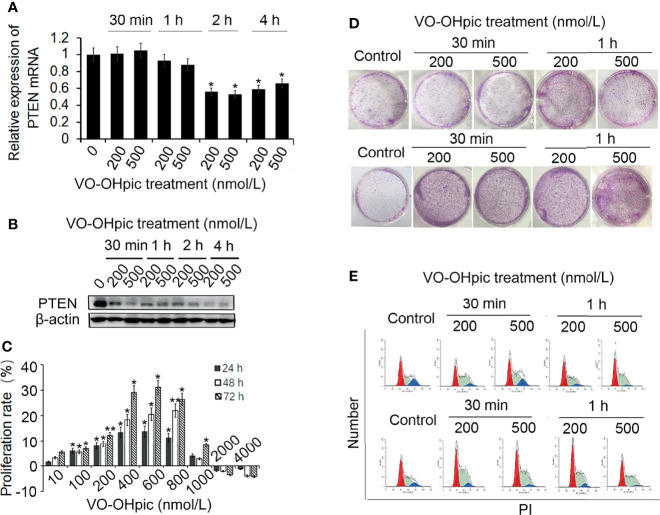
Effects of VO-OHpic on proliferation of 4T1 cells by inhibiting *PTEN* gene expression. 4T1 cells were treated with 200 or 500 nmol/L VO-OHpic for 0.5–4 h and PTEN mRNA expression was detected by real-time quantitative RT-PCR **(A)**, and PTEN protein expression was detected by western blotting **(B)**. **(C)** Effects of different concentrations of VO-OHpic for 4 h on proliferation activity of 4T1 cells. **(D)** Effects of VO-OHpic on cell colony formation and cell cycle phase distribution in 4T1 cells **(E)** after 0.5–4 h treatment. ^*^ *P*<0.05, ^**^ *P*<0.01 compared with the control group.

Treatment of mouse-derived breast cancer 4T1 cells with 200 or 500 nmol/L VO-OHpic for 1–4 h inhibited PTEN mRNA and protein levels, with particularly notable effects after treatment for 2–4 h, especially for PTEN protein (**Figures 1A, B**).

After treatment of 4T1 cells with different concentrations of VO-OHpic for 4 h and culture for 24–72 h, MTT colorimetry showed that 10–800 nmol/L VO-OHpic increased the proliferation activity of 4T1 cells. However, when the concentration of VO-OHpic increased to 1000 nmol/L, the enhancing effect on cell proliferation gradually began to weaken, ultimately leading to inhibition of cell proliferation (**Figure 1C**).

We treated 4T1 cells with 200 or 500 nmol/L VO-OHpic for 0.5–4 h, followed by removal of VO-OHpic and inoculation of the cells into 6-well plates for routine culture. After regular observation, the cells were stained with 0.1% crystal violet to count the number of cell colonies. The number of 4T1 cell colonies was significantly increased after treatment with 200 and 500 nmol/L VO-OHpic, especially after treatment for 2–4 h (**Figure 1D**).

We also detected the cell cycle phase distribution by flow cytometry and showed that the proportion of S phase cells increased to varying degrees, with the most obvious increases of 9.43% and 7.46% at 2 and 4 h, respectively, compared with the control cells (**Figure 1E**), suggesting that VO-OHpic promoted the proliferation of 4T1 cells by inhibiting PTEN.

### Inhibition of PTEN Expression Enhanced Migration and Invasion of 4T1 Cells

**Figure 2:**
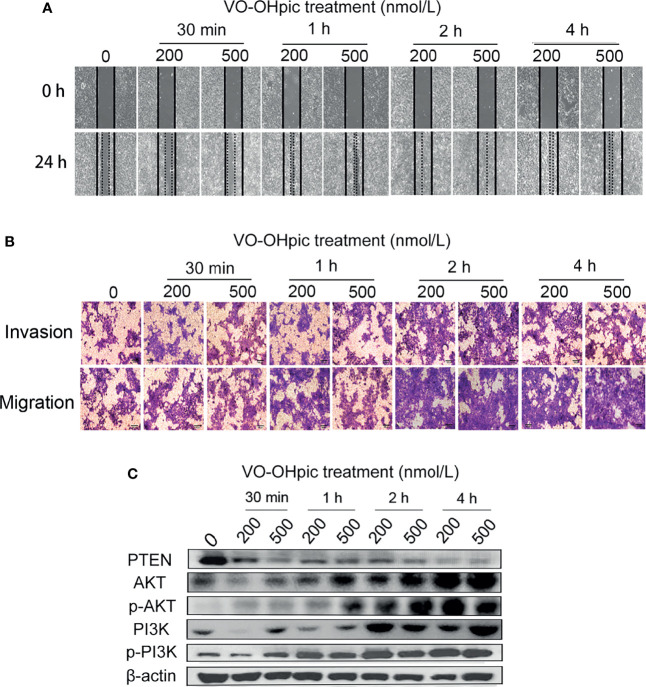
Effect of inhibition of PTEN expression on migration and invasion of 4T1 cells. 4T1 cells were treated with 200 or 500 nmol/L VO-OHpic for 0.5–4 h and their migration and invasion abilities were then detected by cell scratch and transwell assays. **(A)** Cell scratch healing map. **(B)** Observation of cell migration and invasion by the transwell method (original magnification, 100×), and effect of intracellular PTEN on cell proliferation activity **(C)**.

Both 200 and 500 nmol/L VO-OHpic treatment for 30 min-4 h increased the migration rate of 4T1 breast cancer cells and accelerated scratch healing, especially after 2–4 h, according to the cell scratch test (**Figure 2A**). Treatment with 200 or 500 nmol/L VO-OHpic for 30 min–4 h also enhanced the number of 4T1 cells crossing the compartment membrane in transwell assays (**Figure 2B**). These results suggest that inhibition of PTEN expression and activity in mouse breast cancer 4T1 cells by VO-OHpic significantly enhanced their invasion and migration

### PTEN Inhibition Activated PI3K-Akt Signaling Pathway in 4T1 Cells

The PTEN/PI3K/Akt pathway is an important signal-regulation pathway in the body. PTEN can negatively regulate the PI3K/Akt signaling pathway and participate in the physiological and pathological activities of cell proliferation, differentiation and maturation, apoptosis, cycle arrest, invasion, and migration (15, 25, 26). PTEN protein levels in 4T1 cells were inhibited by 200 and 500 nmol/L VO-OHpic to different degrees, while levels of PI3K, p-PI3K, Akt, and p-Akt were significantly increased (**Figure 2C**). These results suggest that inhibition of PTEN promoted the proliferation, migration, and invasion of 4T1 cells by activating the PI3K-Akt pathway.

### Inhibition of PTEN Expression in 4T1 Cells Enhanced Orthotopic Growth and Distant Metastasis *In Vivo*

**Figure 3:**
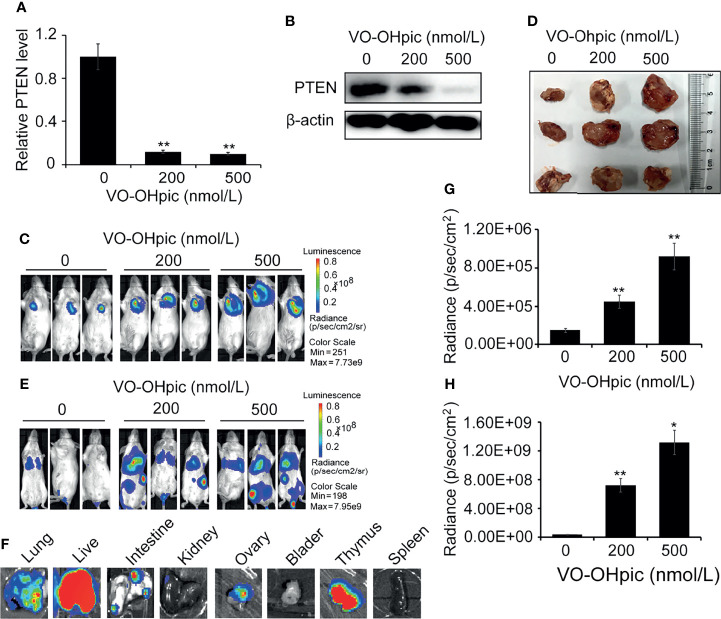
Inhibition of PTEN promoted the growth of orthotopic breast tumors and organ metastases and colonization in mice. PTEN gene **(A)** and protein **(B)** levels in 4T1-luc cells were decreased after treatment with 200 or 500 nmol/L VO-OHpic for 2 h. The above cells were inoculated into the breast fat pad of Balb/c mice to establish an *in situ* tumor model **(C)** and dissected tumor tissues were imaged at 10 days **(D)**. Tumor metastasis models **(F)** were established by injection of the above cells *via* the tail vein, and visceral tumor metastasis was observed at 14 days. Cellular PTEN inhibition by VO-OHpic significantly increased the counts of bioluminescent photons *in situ* breast tumors **(E)** and visceral metastases **(G)**. **(H)** Bioluminescence of some visceral metastases. Compared with 0 nmol/L VO-OHpic group, ^*^ *P*<0.05, ^**^ *P*<0.01.

PTEN was inhibited in 4T1-luc cells by treatment with 200 or 500 nmol/L VO-OHpic for 2 h (**Figures 3A, B**), and the cells were then inoculated into Balb/c mice to establish an orthotopic breast cancer tumor model, or into the tail vein to establish a model of breast cancer metastasis. Tumor growth and metastasis were observed using an IVIS. PTEN function was inhibited in 4T1-luc cells, and the growth rate was faster than that of tumors created by normal PTEN cells, the mean sizes of *in situ* mammary tumors in mice increased from 0.44×0.37 cm^2^ (length×width) of control group to 1.59×1.68 cm^2^ (200 nmol/L) and 1.86×1.72 cm^2^ (500 nmol/L), respectively (**Figures 3C–E**). Organ metastases, including lung, liver, kidney, intestine, bladder, thymus, and ovary metastases, were also significantly increased in mice injected with 4T1-luc cells treated with the above concentrations of VO-OHpic, and tumor bioluminescence was significantly enhanced (**Figures 3F–H**).

### The PTEN Loss of Host Systemic Microenvironment Enhanced the *In Vivo* Growth and Distant Metastasis of 4T1 Breast Cancer Cells

**Figure 4:**
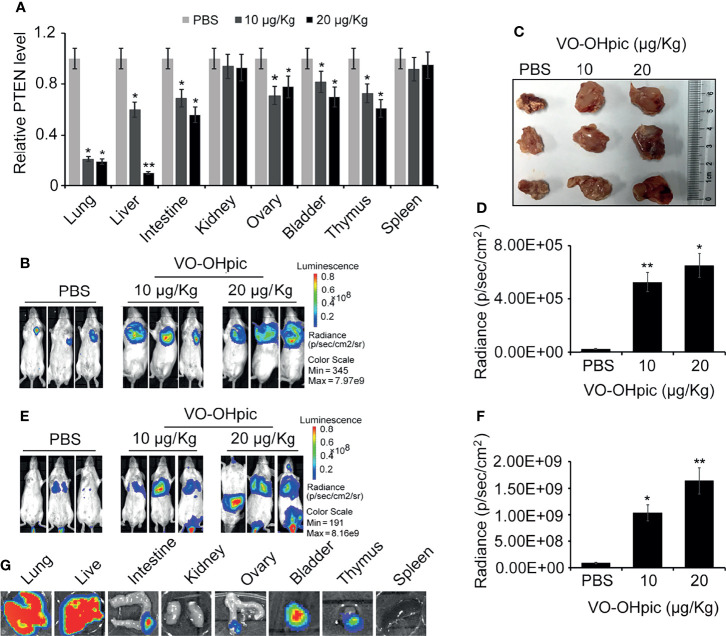
Inhibition of PTEN in mice promoted the metastasis and proliferation of 4T1-luc cells in mice. Expression levels of PTEN mRNA in some organs of Balb/c mice were affected by intraperitoneal injection of 10 or 20 μg/kg VO-OHpic for 1 h, as detected by real-time PCR **(A)**. Routine cultured 4T1-luc cells were inoculated into the breast fat pad of mice treated as above to establish an *in situ* tumor model and tumor formation was observed at 10 days using an IVIS **(B)**. Some exfoliated tumor tissues *in situ* were imaged **(C)**. A metastatic breast cancer model was established by injecting 4T1-luc cells into the tail vein **(E)**. PTEN inhibition by intraperitoneal injection of VO-OHpic *in vivo* significantly increased the counts of bioluminescent photons *in situ* breast tumors **(D)** and visceral metastases **(F)**. Bioluminescence of metastatic tumors in organs including the lung, liver, and intestine were observed using an IVIS **(G)**. Compared with control group, ^*^ *P*<0.05, ^**^ *P*<0.01.

*PTEN* gene levels in the lungs, liver, intestine, ovary, bladder, and other organs were significantly decreased following intraperitoneal injection of 10 or 20 μg/kg VO-OHpic for 1 h (**Figure 4A**). Routine cultured 4T1-luc cells were inoculated into Balb/c mice to establish *in situ* and metastasis breast tumor models. Observation by small-animal *in vivo* imaging showed that inhibition of PTEN *in vivo* increased the growth of mouse breast tumors *in situ*, with increased mean length×width from 0.82×1.01 cm^2^ of control group to 1.41×1.63 cm^2^ (10 μg/kg) and 1.89×1.97 cm^2^ (20 μg/kg), respectively (**Figures 4B–D**), and increased tumor metastasis in distant organs. Bioluminescence of metastatic foci was significantly enhanced (**Figures 4E–G**).

### PTEN-Inhibited 4T1 Breast Cancer Cells in a Systemic PTEN-Deficient Host Microenvironment Showed Greater Proliferation and Metastasis Potential

**Figure 5:**
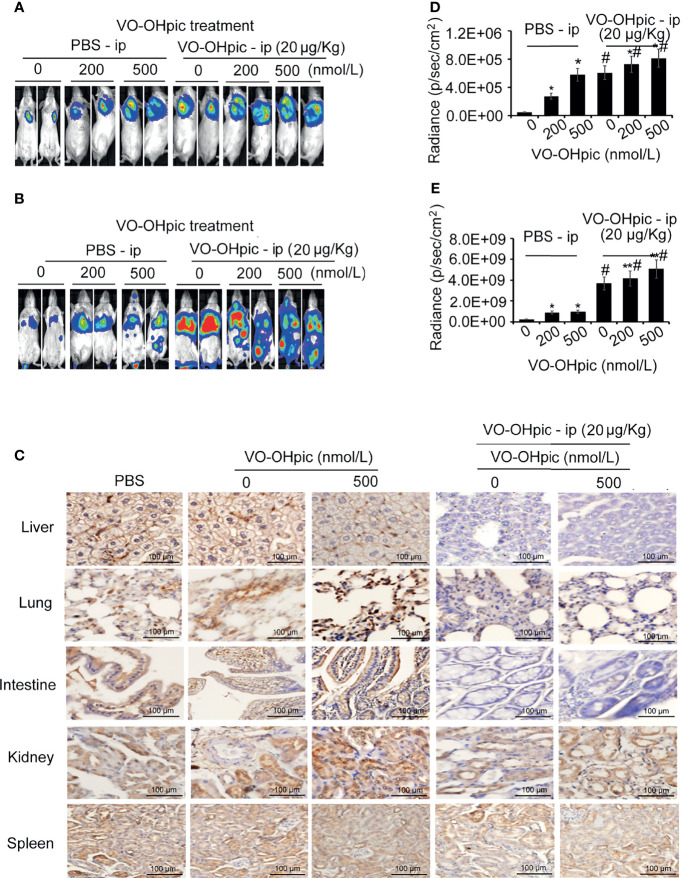
PTEN-deficient breast cancer cells showed greater proliferation and metastasis potential in hosts with low PTEN function. 4T1-luc cells treated with VO-OHpic were inoculated *via* the breast fat pad or tail vein into Balb/c mice intraperitoneally injected with VO-OHpic, to establish a local model **(A)** and an organ metastasis model **(C)**. Flux intensity of breast tumors *in situ* **(B)** and distant metastases **(D)** were significantly increased in mice treated with PTEN inhibition *in vivo* and simultaneous inoculation of PTEN-deficient 4T1-luc cells, compared with mice treated with cellular or *in vivo* PTEN inhibition alone. PTEN protein expression levels in the lung, liver, intestine, and kidney were observed by immunohistochemistry under light microscopy (original magnification, 20×) **(E)**. Compared with 0 nmol/L VO-OHpic group, ^*^ *P*<0.05, ^**^ *P*<0.01. Compared to PBS - ip group, ^#^ *P*<0.01.

Balb/c mice treated with 20 μg/kg VO-OHpic by intraperitoneal injection for 1 h were inoculated with 4T1-luc cells treated with 200 or 500 nmol/L VO-OHpic to establish *in situ* and metastasis breast cancer models, respectively. Proliferation of breast tumors (**Figures 5A, B**) and distant metastases (**Figures 5C, D**) were significantly increased in mice treated with PTEN inhibition *in vivo* and simultaneous inoculation of PTEN-deficient 4T1-luc cells, compared with mice treated with cellular or *in vivo* PTEN inhibition alone (**Figures 5A–D**). Notably PTEN inhibition by intraperitoneal injection of VO-OHpic *in vivo* resulted in larger *in situ* breast tumors, more metastatic organs, and faster growth of transplanted tumors (**Figures 5C, D**, **Table 1**). Immunohistochemical detection showed that organs with more obvious tumor metastasis and faster tumor growth, such as the lung, liver, and intestine, had lower PTEN protein expression levels (**Figure 5E**), while PTEN expression was not significantly changed in the spleen, kidney, and other organs with less metastatic tumor proliferation. These results suggest that inhibition of intracellular PTEN promoted the rapid proliferation of tumor cells, while PTEN inhibition in mouse organs also promoted the colonization and rapid growth of tumor cells in the corresponding organs. Thus PTEN-deficient breast cancer cells were more likely to show accelerated growth and metastases to distant organs if PTEN activity in the body was low.

**Table 1:** Organic metastases of PTEN-inhibited and -uninhibited B16 cells in wild and PTEN-deficient Balb/c mice.

Mouse viscera		Mice / PBS ip			Mice / VO-OHpic ip (20 μg/kg)		
		4T1 / VO-OHpic (nmol/L)			4T1 / VO-OHpic(nmol/L)		
		0	200	500	0	200	500
Liver	NMT	0	3	6	0	3	5
	MTD (L×W cm^2^)		0.32×0.3	0.8×0.52	0.4×0.5	1.23×0.77	1.1×1.4
Lung	NMT	5	8	10	3	10	10
	MTD (L×W cm^2^)	0.14×0.17	0.3×0.17	0.93×0.7	0.4×0.38	1.41×1.25	1.35×1.5
Intestine	NMT	1	2	5	0	6	8
	MTD (L×W cm^2^)		0.33×0.3	0.8×1.3		1.3×1.2	1.4×1.1
Kidney	NMT	0	0	0	0	1	0
	MTD (L×W cm^2^)					0.3×0.4	
Spleen	NMT	0	0	0	0	0	0
	MTD (L×W cm^2^)						
Thymus	NMT	0	0	0		2	3
	MTD (L×W cm^2^)					0.3×0.1	0.2×0.2
Bladder	NMT	0	0	0		1	2
	MTD (L×W cm^2^)					0.2×0.1	0.2×0.2
Ovary	NMT	0	0	0		2	3
	MTD (L×W cm^2^)					0.3×0.17	0.29×0.25

Mean tumor diameter (MTD), L (long), W (wide).

The number of metastatic tumors of mice (NMT).

## Discussion

Breast cancer is a malignant mammary epithelial tumor. Although local breast cancer is rarely fatal, breast tumor cells shed and spread easily throughout the body *via* the blood and lymph circulation, resulting in various potentially lethal metastatic tumors.

The *PTEN* gene is widely involved in the regulation of important signaling pathways including the PI3K/Akt/mTOR, FAK/p130cas, and ERK/MAPK signaling pathways (15, 25–27), and is one of the most important tumor suppressor genes identified to date. Its main functions include inhibiting cell adhesion and migration, inducing cell apoptosis, blocking the cell cycle and cell proliferation, inhibiting the generation of new blood vessels, DNA repair, and regulating normal embryo development, aging, and metabolism (10, 18, 26–29). Abnormal expression or function of PTEN has been closely related to the occurrence and development of various malignant tumors, including endometrial, prostate, liver cancer, colorectal, breast, bladder, stomach, and lung cancers and leukemia (10–14, 19, 20, 22–25). PTEN expression is significantly reduced in many malignant tumors compared with paracancerous tissues/normal cells, especially in highly invasive, highly metastatic, and poorly differentiated cancers, most of which demonstrate *PTEN* mutations or deletions (10–14, 19, 20, 22–25). Migration and invasion abilities are significantly enhanced in tumor cells with *PTEN* deletions or mutations (10–14, 23–29). In addition, PTEN promotes the proliferation, invasion, and metastasis of breast cancer cells, and PTEN levels in metastatic breast cancer cells are much lower than in localized cancer cells (9, 13, 15, 18, 19). However, most studies of the relationship between the tumor suppressor gene *PTEN* and malignant tumors have focused on changes in the expression and function of tumor suppressor genes in the tumor cells themselves, while few reports have considered the effects of the expression and function of tumor suppressor genes such as *PTEN* in the host as a whole, on tumor cell invasion, metastasis, or colonization. In the current study, we treated murine 4T1 breast cancer cells with the highly effective PTEN inhibitor VO-OHpic, and showed that VO-OHpic effectively inhibited PTEN gene and protein expression levels, accompanied by significantly enhanced cell proliferation and increased colony-forming ability, altered the cell cycle distribution, and increased the proportion of S phase cells, suggesting that the rapid proliferation of 4T1 cells might be related to decreased expression or activity of PTEN in the cells.

PI3K is an intracellular phosphatidylinositol kinase with serine/threonine kinase activity (25–28). Its key downstream effector molecule Akt is also a serine/threonine protein kinase. Activation of PI3K promotes the growth and invasion of tumor cells *in vivo* by inactivating various effector molecules to regulate the cell cycle, promote blood vessel growth, and activate cell migration and other related procedures (25–28). The current results showed that inhibition of PTEN gene and protein expression by VO-OHpic significantly accelerated the rate of healing of cell scratches in 4T1 cells. Moreover, the ability of cells to pass through the transwell compartment membrane was enhanced, and the number of cells reaching the lower chamber through the Matrigel was thus significantly increased. These results indicated that the proliferation activity of PTEN-deficient cells was greatly increased, and intercellular adhesion, invasion, and migration were also significantly enhanced. PTEN negatively regulates the classical PI3K/Akt signaling pathway, inhibits cell proliferation, and promotes cell apoptosis. PTEN protein blocks the PI3K/Akt signaling pathway by causing dephosphorylation of the 3’ phosphoric acid of phosphatidylinositol-3, 4, 5-triphosphate, thus maintaining the relative stability of PI3K activity and thereby regulating normal cell growth and apoptosis. In addition, the PI3K/Akt signaling pathway is an important pathway regulating a series of physiological and pathological phenomena, such as cell differentiation, maturation, proliferation, and apoptosis. Deletion of the *PTEN* gene in many primary and metastatic tumors leads to uncontrolled continuous signaling of the PI3K/Akt pathway, which in turn stimulates the cells to keep multiplying. Treatment of 4T1 cells with 200 or 500 nmol/L VO-OHpic effectively inhibited PTEN gene and protein expression in 4T1 cells, and significantly up-regulated expression levels of intracellular Akt, p-Akt, PI3K, and p-PI3K proteins. These results indicate that PTEN protein plays a key role in the negative regulation of the PI3K/Akt signaling pathway. Notably PTEN expression in 4T1 cells was significantly decreased after treatment with VO-OHpic for 1–4 h. Activation of the PTEN/PI3K/Akt signaling pathway significantly enhanced cell proliferation, invasion, and migration, consistent with the results of previous studies.

In this study, we established orthotopic xenograft and metastatic breast tumor models by transplantation of 4T1 cells into BALB/c mice and observed the effects of VO-OHpic interventions using small-animal imaging technology. Inhibition of PTEN at the cellular level *in vitro* or direct inhibition of PTEN *in vivo* by intraperitoneal injection of VO-OHpic led to rapid growth of orthotopic transplanted tumors in mice, with general trends towards faster tumor growth and more liver, lung, intestine, bladder, and other distant metastases in line with increasing concentrations of VO-OHpic, including up to almost 100% of lung metastases. Further detection of PTEN gene and protein expression levels in the transplanted tumors and some organs showed that larger transplanted tumors were associated with lower PTEN expression levels. PTEN expression levels in liver, lung, and intestinal tissues, which are prone to metastasis, were significantly inhibited after intraperitoneal injection of VO-OHpic, especially in liver and lung tissues, with intraperitoneal injection of 20 μg/kg VO-OHpic reducing PTEN expression to about 10%–19% of that in the normal control group. Notably, intraperitoneal injection directly inhibited PTEN expression in the internal organs, with lower organ levels associated with a higher metastasis rate, wide implantation area, and fast proliferation of metastatic tumors in the corresponding organs. While the changes in PTEN gene and protein levels in the kidney and spleen were not significant, no colonization or growth of tumor cells generally occurs in these organs, suggesting that the expression and function of the tumor suppressor gene *PTEN* in the tumor-bearing host itself, especially in the microenvironment of the organ, directly affects the invasion, metastasis, colonization, and proliferation of tumor cells. These results demonstrate that a metastatic tumor microenvironment is one in which tumor cells can directly contact, grow, and survive. It can provide essential nutrients and energy for tumor growth, but also enables the metastatic tumor cells to adapt to the new dynamic microenvironment and participate in regulating the signaling pathways related to tumor development. The tumor microenvironment thus aids the growth and proliferation of tumor cells, as well as further invasion and metastasis.

The role of PTEN in tumor-microenvironment cells and its effect on tumor cells have received great attention in recent years. About 25% of patients with breast cancer had loss of PTEN expression in stromal fibroblasts (29–32). Clinical studies also suggested that patients with breast, prostate, pancreatic, and endometrial cancers with PTEN-deficient stromal fibroblasts had a poor prognosis (22, 23, 25, 29–31, 33). Inactivation of *PTEN* in mouse breast stromal fibroblasts accelerated the malignant transformation of breast epithelial cells, and *PTEN* knockout in stromal fibroblasts promoted extracellular matrix remodeling, tumor angiogenesis, proliferation, invasion, metastasis, and other events, and mediated therapeutic resistance in mice with breast cancer (30, 31). Previous studies suggested that the severity and tissue selectivity of the disease in tumor cells and the tumor microenvironment were determined by PTEN protein level rather than *PTEN* gene mutation. Slight changes in PTEN protein levels can have major impacts on oncogenic signaling pathways, and its sensitivity is tissue-specific.

We inhibited the expression and activity of PTEN in host organs by intraperitoneal injection of VO-OHpic. Both local proliferation and distant metastases were significantly enhanced, and the ability of tumor cells to metastasize in each organ was related to the PTEN level in the organ. These results suggest that a low level or deletion of PTEN in the microenvironment of metastatic target organs promotes the colonization by metastatic breast cancer cells and the formation of metastatic tumors. However, use of the chemical inhibitor VO-OHpic to inhibit systemic PTEN in mice may be associated with problems related to the non-specific unexpected effects of chemical substances and persistence of effects. It is therefore necessary to determine the detailed effects of PTEN on breast cancer metastasis in relation to the host as a whole and the organ tissue microenvironment, and to establish a mouse model of complete *PTEN* knockout. However, it is difficult to establish such a model due to the embryo-lethal nature of *PTEN* total knockout, and only tissue- or organ-specific conditional knockout strategies can be adopted (27). However, if *PTEN* is knocked out in only one or several specific tissues or organs, normal expression of *PTEN* in the other tissues, including immune system cells, will affect the microenvironment of the knocked-out tissues and organs, thus affecting the reliability of the conclusions. We aim to conduct future studies to explore targeted ways of solving these problems, to clarify the molecular mechanism by which *PTEN* regulates the co-evolution of metastatic cancer cells and the metastatic microenvironment, and to establish a strategy to reduce the invasion and metastasis of breast cancer cells by enhancing the function of tumor suppressor genes in tumor-bearing organisms.

The current findings showed that decremental loss of *PTEN* expression and activity in the host by *in vivo* injection of VO-OHpic significantly enhanced the growth of orthotopic breast cancer and its metastasis to distant organs, with the metastasis intensity in each organ being negatively related to the PTEN expression level in the organ. These facts suggest that a systemic loss of or decrease of PTEN in the microenvironment of metastatic target organs promotes the implantation of metastatic breast cancer cells and the formation of metastatic tumors. However, the *in vivo* use of the chemical PTEN inhibitor VO-OHpic may have unexpected non-specific chemical effects, as well as causing persistent inhibition. The ideal strategy for investigating the precise effect of PTEN in the microenvironment of specific tissues or organ or in the body as a whole on the metastasis of breast cancer would involve the creation of a conventional knockout mouse model; however, this is not possible because of the embryo-lethality of systemic *PTEN* knockout. Thus only tissue- or organ-specific conditional knockout strategies have been available to date; however, *PTEN* knockout in only one or several specific tissues or organs may be affected by PTEN from non-knockout tissues or organs, especially the immune system, with potential implications for the reliability of the study conclusions. We are conducting ongoing studies to explore targeted methods or models for elucidating the mechanisms by which PTEN regulates the co-evolution of metastatic cancer cells and the metastatic microenvironment, and to find an effective means of preventing the invasion and metastasis of human breast cancer cells *via* systemic elevation of tumor suppressor gene function in the tumor-bearing host.

## Data Availability Statement

The raw data supporting the conclusions of this article will be made available by the authors, without undue reservation.

## Ethics Statement

The animal study was reviewed and approved by Laboratory Animal Science and Technology Management Committee of the School of Basic Medicine of Lanzhou University.

## Author Contributions

All authors listed have made a substantial, direct, and intellectual contribution to the work and approved it for publication.

## Funding

This work was mainly supported by Natural Science Fund of Gansu province, China (20JR5RA269, 20JR5RA281 and 18JR3RA291) and National Natural Science Foundation of China (NO. 31701206).

## Conflict of Interest

The authors declare that the research was conducted in the absence of any commercial or financial relationships that could be construed as a potential conflict of interest.

## Publisher’s Note

All claims expressed in this article are solely those of the authors and do not necessarily represent those of their affiliated organizations, or those of the publisher, the editors and the reviewers. Any product that may be evaluated in this article, or claim that may be made by its manufacturer, is not guaranteed or endorsed by the publisher.
